# Recurrent non-suicidal self-injury in depressed youth with mixed features: a 6-month prospective cohort study

**DOI:** 10.1186/s13034-025-01006-z

**Published:** 2025-12-09

**Authors:** Kunrong Lin, Yuhang He, Xue Zeng, Jie Zhang, Yufen Ou, Hongbo He

**Affiliations:** 1 The Affiliated Brain Hospital, Guangzhou Medical University, Guangzhou, China; 2 Guangdong Engineering Technology Research Center for Translational Medicine of Mental Disorders, Guangzhou, China; 3 Guangdong Mental Health CenterGuangdong Provincial People’s Hospital, Guangdong Academy of Medical Sciences, Southern Medical University, Guangzhou, 510030 China

**Keywords:** Mixed features, Non-suicidal self-injury (NSSI), Major depressive episode (MDE), Adolescents and young adults, Recurrent self-injury

## Abstract

**Objective:**

This study aimed to examine whether mixed features during a current major depressive episode (MDE) are associated with increased risk of non-suicidal self-injury (NSSI) among adolescents and young adults, focusing on both first-onset and recurrent NSSI during a 6-month follow-up period.

**Method:**

A total of 713 individuals aged 13–25 years with current MDE were recruited, including 233 with mixed features. NSSI was assessed at baseline and at 1-, 3-, and 6-month follow-ups. Multiple imputation was used to handle missing data (*n* = 626). Kaplan–Meier and Nelson–Aalen estimators were applied to visualize time-to-event and cumulative risk curves. Cox regression assessed first-onset NSSI, and Andersen–Gill models estimated the risk of repeated events. Rubin’s rules were used to pool estimates across imputed datasets. Sensitivity analyses were performed using complete-case data after multiple imputation, while subgroup analyses were conducted using stratified models.

**Results:**

Participants with mixed features were more likely to be female and to report a shorter illness duration and aggression history. Mixed features were associated with earlier NSSI onset (*p* = .010) and higher cumulative risk (*p* < .001). Although no significant association was found with first-onset NSSI, mixed features significantly predicted recurrent NSSI in both imputed (HR = 1.35, *p* = .045) and complete-case models (HR = 1.58, *p* < .001). The effect was stronger among first-episode cases and those with illness duration < 6 months.

**Conclusion:**

Mixed features in adolescent and young adult MDE may serve as a predictor of recurrent NSSI. Early identification and tailored monitoring strategies are warranted to reduce self-injury risk.

**Supplementary Information:**

The online version contains supplementary material available at 10.1186/s13034-025-01006-z.

## Introduction

 Non-suicidal self-injury (NSSI) is highly prevalent among youth and carries substantial clinical significance [1, 2], particularly in individuals with major depressive disorder (MDD) [3, 4]. Epidemiological data indicate that approximately 30% to 60% of youth with MDD report engaging in NSSI [5]. Those who self-injure exhibit markedly elevated risk for suicide and often develop repetitive or chronic self-harming behaviors, leading to significant functional impairment and placing a considerable burden on families and healthcare systems [6, 7].

Mixed features, as defined in the Diagnostic and Statistical Manual of Mental Disorders, Fifth Edition (DSM-5), refer to the presence of subthreshold manic symptoms during a depressive episode that do not meet full criteria for mania or hypomania [8–10]. Approximately 23.8% of individuals with depressive disorders meet criteria for mixed features [11], and this subgroup has been consistently associated with increased risk of suicide attempts [12]. Furthermore, previous studies have indicated that youth with mixed features often exhibit increased emotional arousal and poor impulse control, both of which have been consistently associated with NSSI [13]. Elevated emotional arousal and impaired impulse control may contribute to NSSI by intensifying emotional distress and diminishing inhibitory capacity [14, 15].

Despite these associations, few studies have systematically investigated the impact of mixed features on NSSI, particularly in youth populations. Moreover, there is a paucity of longitudinal data on the developmental trajectories of self-injurious behaviors in this group [16, 17]. To address this gap, the present multicenter prospective study aimed to examine whether the presence of mixed features at baseline predicts the occurrence of NSSI within a six-month follow-up period. Elucidating this potential relationship may contribute to earlier identification of at-risk youth and inform the development of developmentally appropriate, targeted intervention strategies.

## Methods

Participants were recruited from the South China Adolescent Depression Cohort, a multicenter prospective longitudinal study coordinated by the Affiliated Brain Hospital of Guangzhou Medical University in collaboration with multiple psychiatric institutions across South China. Recruitment took place between March 1, 2022, and March 31, 2024.

Eligible participants were aged 13–25 years, met DSM-5 criteria for major depressive disorder (MDD), and were currently experiencing a major depressive episode (MDE). Individuals were excluded if they had a prior diagnosis of schizophrenia, bipolar disorder, intellectual disability, or other severe psychiatric conditions, or if they exhibited cognitive or language impairments that interfered with assessment completion.

The study protocol was approved by the Ethics Committee of the Affiliated Brain Hospital of Guangzhou Medical University (Approval No. 2022-031), and written informed consent was obtained from all participants. For minors (< 18 years), consent was additionally obtained from a parent or legal guardian.

### Measure

#### Baseline characteristics

Baseline data included sociodemographic variables (e.g., age, sex, and romantic relationship status), clinical variables (e.g., illness duration categorized as < 6 months, 6–24 months, or > 24 months; first vs. recurrent episode), physical comorbidity status (none, current, or past), and history of aggressive behavior (no aggression vs. current or past aggression). These variables were collected at baseline through structured clinical interviews and standardized self-report forms.

### Assessment of mixed features

Mixed features were defined in accordance with the DSM-5-TR specifier for major depressive episodes, which requires the presence of at least three manic or hypomanic symptoms occurring nearly every day during the majority of days in a depressive episode, without meeting full criteria for a manic or hypomanic episode (American Psychiatric Association, 2022). Assessments were independently conducted by two senior psychiatrists with expertise in mood disorders, using clinical interviews, observational data, and collateral reports. Symptoms assessed included elevated or expansive mood, inflated self-esteem or grandiosity, increased talkativeness, flight of ideas, increased energy or goal-directed activity, involvement in risky activities, and decreased need for sleep. Diagnostic discrepancies were resolved through consensus discussion. Participants were subsequently categorized into groups with and without mixed features.

### Assessment of NSSI

In the present study, NSSI was assessed using the Brief Non-Suicidal Self-Injury Assessment Tool (BNSSI-AT) [18, 19], a validated instrument widely used to evaluate both primary and secondary forms of self-injurious behavior. This tool captures detailed information regarding the frequency, methods, functions, and anatomical sites of injury, thereby allowing for clear differentiation between NSSI and suicidal behavior.

Specifically, Items 1 and 2 were used to determine whether participants had ever engaged in self-injurious acts and to identify the specific methods involved. Item 3 addressed the underlying motivations for such behavior. If participants selected “as a way to attempt suicide” or “a suicide attempt” in Item 3, or responded affirmatively to Item 4 (“You mentioned deliberately hurting yourself to practice or attempt suicide. Was this the main reason for your self-injury?”), they were excluded from the NSSI group.

At baseline, participants were asked to report any prior history of NSSI. To monitor new and recurring self-injury, assessments were repeated at 1-, 3-, and 6-month follow-up points, using the same instrument. All follow-up evaluations were conducted remotely via WeChat, structured telephone interviews, or a secure digital platform. In line with best practices for clinical reliability, all assessments were conducted by psychiatrists who had received standardized training in the use of the BNSSI-AT.

### Statistical analysis

#### Descriptive and group comparisons

All variables were treated as categorical. Continuous variables were handled as follows: age was dichotomized at the clinically meaningful cutoff of 18 years to improve interpretability, whereas illness duration was categorized into three groups: less than 6 months, 6 to 24 months, and more than 24 months. Based on the presence or absence of mixed features, participants were divided into two groups. Group comparisons of baseline characteristics were conducted using chi-square tests.

To further explore the association patterns among variables, Cramér’s V coefficients were calculated. Cramér’s V is an unbiased measure used to estimate effect sizes between categorical variables. Pairwise associations were visualized using a triangular correlation heatmap, which facilitated the identification of potential multicollinearity and the selection of appropriate covariates for multivariable modeling. This approach was intended to improve the robustness and explanatory power of the final models.

### Missing data handling

Missing data patterns were evaluated using Little’s Missing Completely at Random (MCAR) test [20, 21], which supported the assumption of data missing completely at random (*p* >.05). To ensure reliable imputation, multiple imputation was restricted to participants who completed at least two of the three follow-up assessments. Individuals with missing data at all follow-up time points were excluded from the imputation process.

The imputation model included binary indicators of NSSI at 1-, 3-, and 6-month follow-ups, along with baseline covariates that may be related to missingness or outcomes, such as mixed features status, sex, episode type, history of aggression, lifetime self-harm, age, and illness duration.

Binary logistic regression was used to impute the categorical NSSI outcome variables. Multiple imputation by chained equations (MICE) was applied [22], using five imputed datasets, with a maximum of 50 iterations and a fixed random seed to ensure reproducibility. Importantly, imputation was limited to binary outcome variables at fixed time points; survival times were not imputed, and no assumptions related to censoring were introduced in this process.

### Modeling of NSSI outcomes

Time-to-event distributions and cumulative risk of NSSI were visualized using Kaplan–Meier survival curves and Nelson–Aalen cumulative hazard functions, based on the first imputed dataset. Group differences in survival distributions between participants with and without mixed features were assessed using the log-rank test [23].

Cox proportional hazards models were used to estimate the effect of mixed features on the time to the first occurrence of NSSI during the follow-up period. This model evaluated the relative hazard of initial self-injury events associated with mixed features, while adjusting for potential confounding effects of baseline covariates. Considering that participants may engage in NSSI more than once during the follow-up period, Andersen–Gill (AG) models were employed to examine the risk of recurrent NSSI events. As an extension of the Cox model, the AG model allows for multiple event entries per individual, thereby providing a more comprehensive assessment of the dynamic risk of repeated self-injury [24, 25]. The AG model accounts for within-subject correlation between events, thereby providing a more comprehensive assessment of the dynamic risk of repeated self-injury. Additionally, AG analyses were conducted using complete-case data to further validate the accuracy and robustness of the model estimates.

Both Cox and Andersen–Gill models included baseline covariates significantly associated with NSSI, including sex, age category, episode type, history of aggression, and lifetime NSSI. The proportional hazards assumption was tested using Schoenfeld residuals [26]. For variables violating this assumption, stratified Cox models were applied to allow for non-proportional hazards across strata [23, 27].

Extending the Andersen–Gill model framework, stratified analyses were performed to investigate the influence of mixed features on the risk of non-suicidal self-injury (NSSI) within distinct subgroups. In each model, baseline history of self-harm was included as a covariate alongside mixed features. Interaction terms between mixed features and stratification variables were incorporated to assess the statistical significance of effect modification across strata. To visually summarize these results, a forest plot was generated displaying the magnitude and confidence intervals of the effect of mixed features on NSSI risk within each subgroup.

### Software and statistical thresholds

All analyses were conducted in R (version 4.4.2), and two-sided p-values less than 0.05 were considered statistically significant.

## Results

### Sample characteristics

A total of 1,631 participants were enrolled in the overall cohort study. Of these, 580 individuals were excluded from the present analysis as they either fell outside the specified age range of 13 to 25 years, did not meet the diagnostic criteria for a current major depressive episode, or had missing baseline data. Additionally, 338 participants failed to complete any follow-up assessments and were therefore excluded from the longitudinal analyses. Consequently, 713 adolescents and young adults were included in the current study. Among these participants, 233 (32.7%) fulfilled the DSM-5 specifier criteria for mixed features during their depressive episode. Regarding follow-up assessments, 626 participants completed evaluations at least at two time points, and 476 participants completed assessments at all three time points (Supplementary Materials).

Baseline characteristics stratified by mixed features status are summarized in Table 1. Compared to participants without mixed features, those with mixed features were significantly more likely to be female (82.8% vs. 74.6%, χ² = 5.62, *p* = .018), exhibit recurrent episodes (69.5% vs. 43.4%, *p* < .001), have longer illness duration (*p* < .001), and report higher rates of current or past aggressive behavior (20.6% vs. 9.8%, *p* < .001). Furthermore, a significantly greater proportion of individuals in the mixed features group reported a lifetime history of NSSI (71.7% vs. 57.9%, *p* < .001). No statistically significant differences were observed between groups regarding age category, romantic relationship status, or physical comorbidities. During follow-up, NSSI occurrence at different time points is presented in Table 2. Participants with mixed features showed significantly higher rates of NSSI at 0–1 month (30.5% vs. 22.7%, χ² = 4.61, *p* = .032) and 3–6 months (28.3% vs. 15.4%, χ² = 10.72, *p* = .001) compared with those without mixed features, whereas no significant difference was observed at 1–3 months (23.2% vs. 19.3%, χ² = 0.98, *p* = .323).

**Table 1:** Baseline sociodemographic and clinical profile by mixed features status

Variable	MDD With Mixed Features(*n* = 233)	MDD Without Mixed Features(*n* = 480)	Statistic	p_value
Age, years (mean ± SD)	18.4 ± 3.23	18.78 ± 3.05	t = 1.47	0.143
Age group, n (%)				
<18 years	103 (44.2%)	187 (39.0%)	χ² = 1.58	0.209
≥ 18years	130 (55.8%)	293 (61.0%)		
Gender, n (%)				
Male	40 (17.2%)	122 (25.4%)	χ² = 5.62	0.018
Female	193 (82.8%)	358 (74.6%)		
Romantic Relationship Status, n (%)				
In a relationship	43 (18.5%)	102 (21.2%)	χ² = 0.59	0.441
Not in a relationship	190 (81.5%)	378 (78.8%)		
Illness duration, months (mean ± SD)	35.83 ± 28.83	26.27 ± 24.96	t=-4.56	< 0.001
Illness duration category, n (%)				
< 6 months	26 (11.2%)	101 (21.0%)	χ² = 23.54	< 0.001
6–24 months	77 (33.0%)	199 (41.5%)		
> 24 months	130 (55.8%)	180 (37.5%)		
First episode, n (%)				
Yes	88 (37.8%)	280 (58.3%)	χ² = 25.75	< 0.001
No	145 (62.2%)	200 (41.7%)		
Physical Comorbidity, n (%)				
No	151 (64.8%)	341 (71.0%)	χ² = 2.96	0.228
Current	49 (21.0%)	80 (16.7%)		
Past	33 (14.2%)	59 (12.3%)		
Aggressive Behavior Status, n (%)				
No Aggression	185 (79.4%)	433 (90.2%)	χ² = 14.95	< 0.001
Current/Past Aggression	48 (20.6%)	47 (9.8%)		
NSSI history, n (%)				
Yes	167 (71.7%)	278 (57.9%)	χ² = 12.07	< 0.001
No	66 (28.3%)	202 (42.1%)		

**Table 2:** Self-harm within different time frames in youth with and without mixed features

Time frame of self-harm	Non-mixed	Mixed	χ²	df	*P*
0–1 month	109 (22.7%)	71 (30.5%)	4.61	1	0.032
1–3 months	80 (19.3%)	43 (23.2%)	0.98	1	0.323
3–6 months	53 (15.4%)	45 (28.3%)	10.72	1	0.001

Data are presented as n (%). Group differences were analyzed using the chi-square test

### Missing data and model assumptions

Little’s MCAR test indicated that missing data were missing completely at random (*p* = .508), thus justifying the use of multiple imputation. The proportional hazards assumption was assessed using Schoenfeld residuals, revealing that all covariates met the assumption except for lifetime NSSI history in recurrent self-harm models (*p* < .05). This violation was addressed by applying stratified Cox regression with strata defined by lifetime self-harm status.

### Time to first NSSI during follow-up

**Fig. 1:**
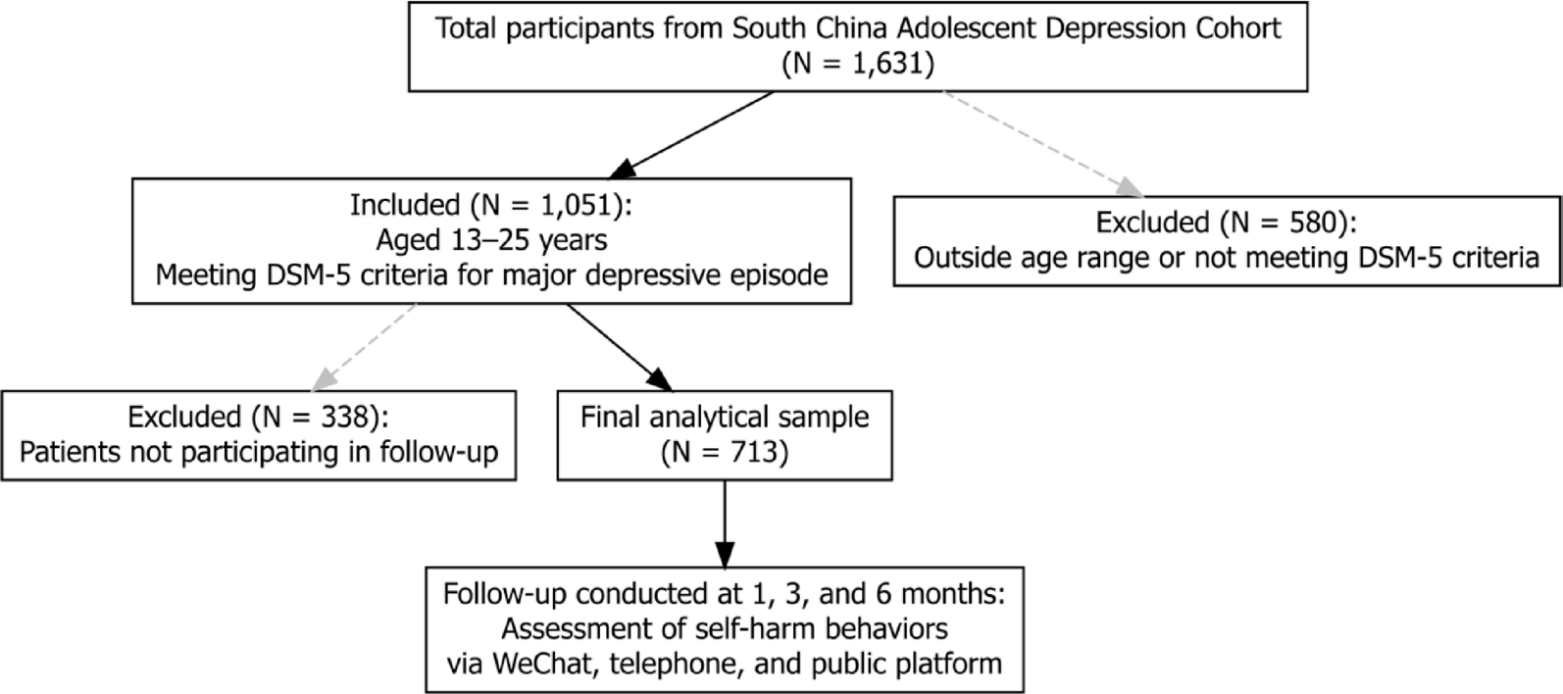
Participant Inclusion and Exclusion Flowchart

**Fig. 2:**
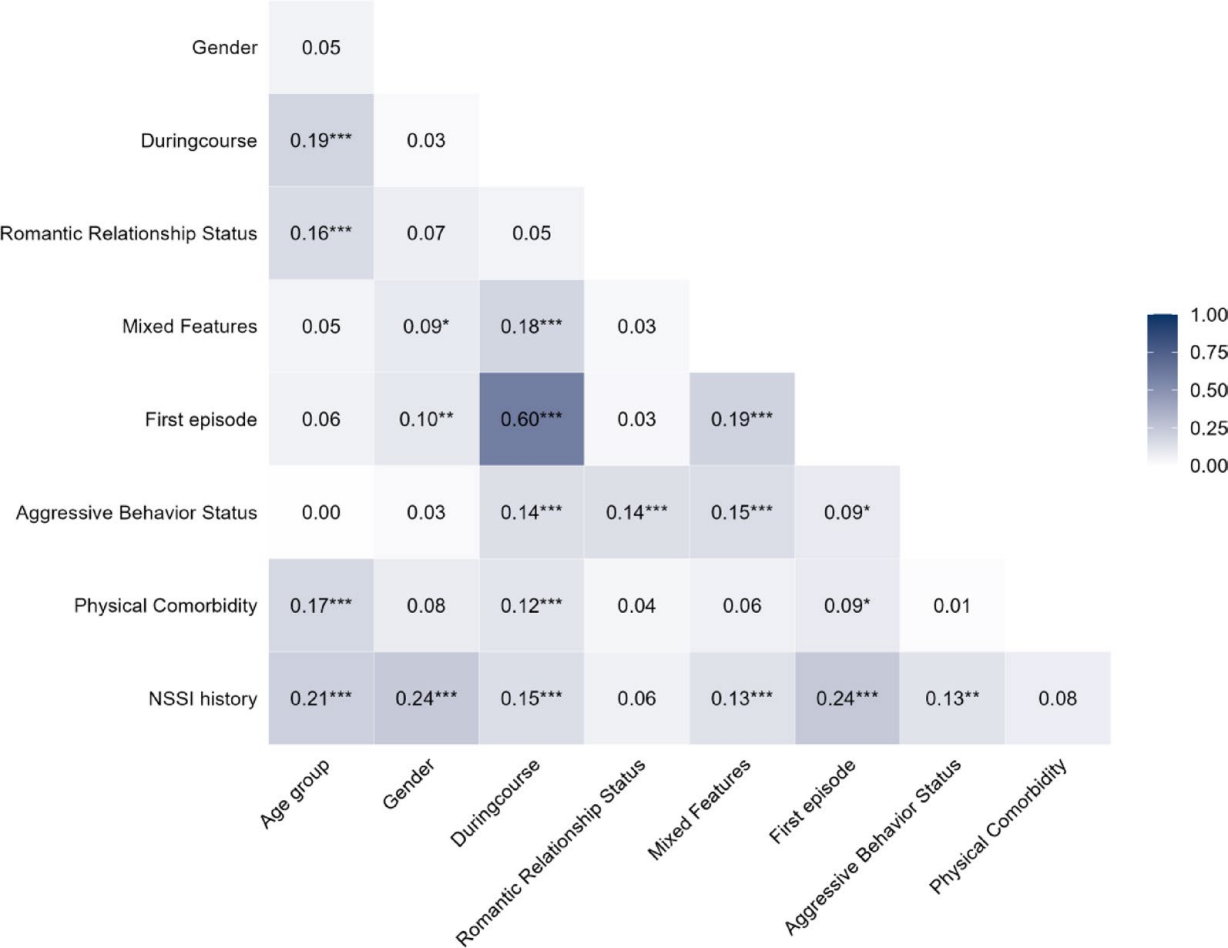
Cramér’s V Heatmap of Pairwise Associations Among Sociodemographic and Clinical Variables. This triangular heatmap presents Cramér’s V coefficients for all pairwise associations between key categorical variables. Only the lower triangle is displayed for clarity. Variables include age group, gender, duration of illness, romantic relationship status, mixed features status, first-episode status, history of aggression, physical comorbidities, and NSSI history. Each tile shows the effect size and significance. Darker blue shades reflect stronger associations. Statistical significance is denoted as follows: *p* < .05*, *p* < .01 **, *p* < .001***

Fig. 1 Kaplan–Meier survival analysis based on multiply imputed data revealed that participants with mixed features experienced significantly shorter time to first NSSI compared to those without mixed features (log-rank *p* = .010; Fig. 2a). Nevertheless, this effect did not reach statistical significance in the Cox proportional hazards model (HR = 1.27, 95% CI = 0.96–1.68, *p* = .098). Compared to participants under the age of 18, those aged 18 years or older exhibited a significantly lower hazard of first-onset NSSI during follow-up (HR = 0.68, 95% CI = 0.51–0.92, *p* = .012). In contrast, a lifetime history of NSSI was associated with a substantially increased risk of NSSI onset (HR = 4.26, 95% CI = 2.92–6.22, *p* < .001) (Table 3).

**Table 3:** Cox proportional hazards regression results for first nonsuicidal Self-Injury (Based on multiply imputed Data)

Variable	Level	OR	CI	*p*
Mixed Features(Ref = Without)				
	With	1.27	(0.96–1.68)	0.098
Gender(Ref = male)				
	Female	1.41	(0.96–2.08)	0.082
Age(Ref = <18 years)				
	≥ 18 years	0.68	(0.51–0.92)	0.012
First episode(Ref = Yes)				
	No	1.07	(0.77–1.49)	0.705
Aggressive Behavior Status(Ref = No)				
	Current/Past	0.94	(0.65–1.34)	0.719
Duringcourse(Ref = < 6 months)				
	6–24 months	0.91	(0.6–1.36)	0.634
	> 24 months	0.72	(0.45–1.17)	0.188
NSSI history(Ref = No)				
	Yes	4.26	(2.92–6.22)	< 0.001

This Cox proportional hazards model was used to examine factors associated with the first suicide attempt, defined as the earliest occurrence of self-harm across three follow-up time points (1, 3, or 6 months). Results are presented as hazard ratios (HR) with 95% confidence intervals (CI). Age was dichotomized at 18 years; illness duration was grouped as < 6, 6–24, and > 24 months. Mixed features, first episode status, aggressive behavior, and lifetime history of self-harm were treated as binary variables

### Recurrent NSSI during follow-up

**Fig. 3:**
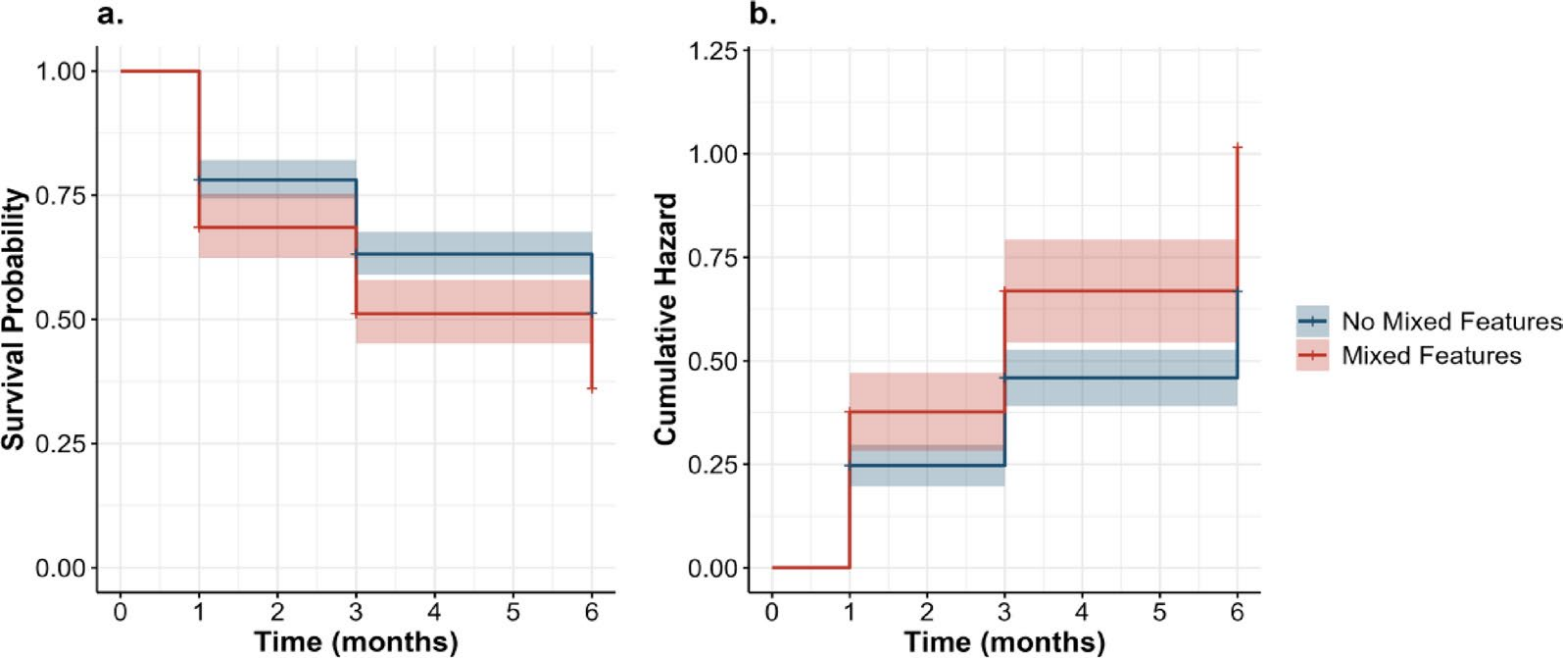
Survival and Cumulative Hazard Curves by Mixed Features. **a** Kaplan–Meier survival curves comparing patients with and without mixed features during the follow-up period. The log-rank test indicated a significant difference between groups, (*P* = .010). **b** Nelson–Aalen cumulative hazard curves stratified by time interval, illustrating a higher cumulative risk for patients with mixed features. The Cox regression model revealed a highly significant group difference, (*P* < .001)

The Nelson–Aalen cumulative hazard curves (Fig. 3b) indicated a higher cumulative risk of recurrent NSSI among participants with mixed features. The log-rank test indicated a highly significant difference in survival distributions between the two groups (*p* < .001). Andersen–Gill recurrent event models demonstrated that mixed features were significantly associated with an increased risk of repeated NSSI in the multiply imputed dataset (HR = 1.35, 95% CI = 1.01–1.80, *p* = .045) and in the complete-case dataset (HR = 1.58, 95% CI = 1.24–2.02, *p* < .001). Additionally, female sex was associated with a higher risk of NSSI recurrence (HR range = 1.50–1.57, *p* < .05), while participants aged 18 years or older had a significantly lower risk of recurrent NSSI compared to those under 18 (HR range = 0.65–0.76, *p* < .05). In contrast, episode type, history of aggression, and illness duration were not significantly associated with NSSI recurrence in any of the tested models (see Table 4).

**Table 4:** Andersen–gill models for recurrent NSSI during 6-month follow-up: sensitivity analyses using multiple imputation and complete-case approaches

		Multiple Imputation		Complete-case Analysis	
Variable	Level	HR (95% CI)	P-value	HR (95% CI)	P-value
Mixed Features(Ref = Without)					
	With	1.35 (1.01–1.80)	0.045	1.58 (1.24–2.02)	<0.001
Gender(Ref = male)					
	Female	1.50 (1.03–2.19)	0.033	1.57 (1.11–2.23)	0.011
Age(Ref = <18 years)					
	≥ 18 years	0.70 (0.53–0.93)	0.014	0.76 (0.60–0.96)	0.024
First episode(Ref = Yes)					
	No	0.89 (0.67–1.20)	0.448	0.92 (0.68–1.24)	0.578
Aggressive Behavior Status(Ref = No)					
	Current/Past	1.11 (0.77–1.60)	0.577	0.98 (0.72–1.35)	0.916
Duringcourse(Ref = < 6 months)					
	6–24 months	0.99 (0.66–1.50)	0.974	0.99 (0.69–1.42)	0.972
	> 24 months	0.78 (0.50–1.23)	0.285	0.69 (0.45–1.05)	0.086

*HR* Hazard Ratio, *CI* Confidence Interval, Ref = Reference group. Both models employed Andersen–Gill estimations. Model 1 utilized multiply imputed data (*n* = 626), while Model 2 was conducted using complete-case data (*n* = 476)

**Fig. 4:**
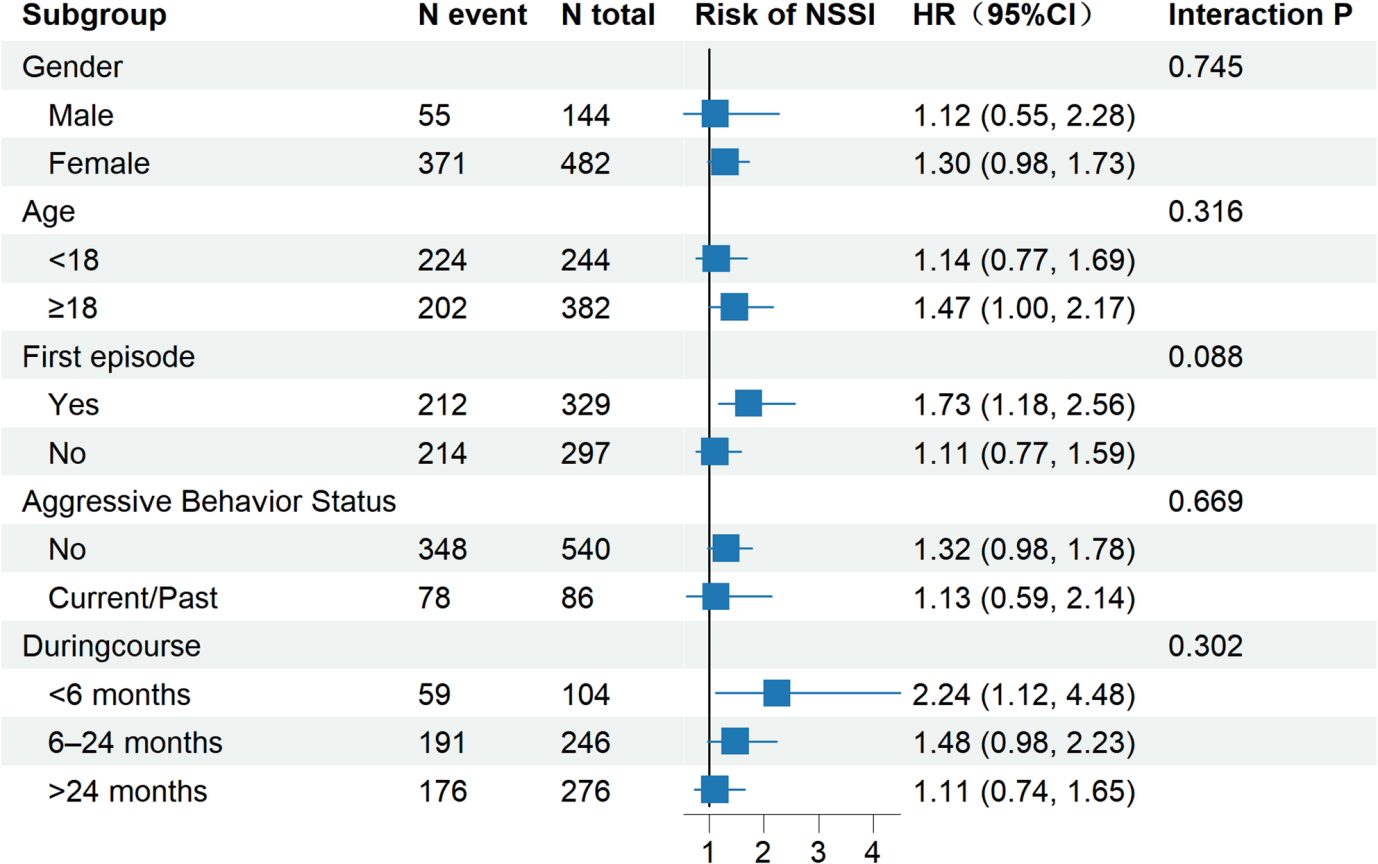
Forest plot of stratified hazard ratios for mixed features predicting self-injurious behavior over 6 months. Forest plot displaying hazard ratios (HRs) and 95% confidence intervals (CIs) for the association between mixed features and self-injurious behavior, stratified by sex, age group, illness duration, first episode status, and aggressive behavior status. Models were adjusted for lifetime self-harm history (stratified) and accounted for clustering by participant ID. Interaction P-values indicate the statistical significance of the interaction between mixed features and each stratifying variable

Based on the stratified analysis, the association between mixed features and the risk of NSSI was significant in certain subgroups. Specifically, participants experiencing their first episode (HR = 1.73, 95% CI: 1.18–2.56) and those with a duration of illness less than 6 months (HR = 2.24, 95% CI: 1.12–4.48) showed a significant relationship. The association in the age group ≥ 18 years approached significance (HR = 1.47, 95% CI: 1.00–2.17). No statistically significant associations were observed in subgroups defined by sex (male HR = 1.12; female HR = 1.30), age < 18 years, recurrent episode status, history of aggression, or illness duration of 6–24 months and > 24 months. Interaction tests across all stratification variables yielded p-values > 0.05, indicating no significant effect modification and suggesting a consistent impact of mixed features on NSSI risk across different strata (Fig. 4).

## Discussion

This study utilized data from a large multicenter cohort in South China to systematically examine the predictive role of mixed features on the risk of recurrent NSSI among youth with depression. Findings demonstrated that mixed features were significantly associated with an increased risk of recurrent NSSI within a six-month follow-up period. This association remained robust across both multiple imputation and complete-case analyses. Stratified analyses further revealed that although mixed features consistently tended to elevate NSSI risk across subgroups, the association was particularly pronounced among individuals experiencing their first depressive episode and those with an illness duration of less than six months.

Although KM survival analysis indicated an earlier onset of NSSI among individuals with mixed features, this association did not reach statistical significance in Cox proportional hazards modeling, suggesting that mixed features may have limited independent prognostic value for incident NSSI. Notably, a prior history of NSSI emerged as the most robust predictor of new-onset NSSI during the follow-up period, underscoring the central importance of behavioral history in risk assessment [28]. In contrast, mixed features were consistently and significantly associated with NSSI recurrence, with hazard ratios ranging from 1.35 to 1.58 across both multiple imputation and complete-case AG models. These findings raise the possibility that mixed features may play a causal role in the persistence and chronicity of self-injurious behaviors.

Our findings expand the current literature on mixed features by shifting the focus from bipolar disorder to depressive episodes. Prior research has primarily examined mixed features in relation to bipolar conversion [29], treatment outcomes [30], and functional impairment [31, 32], with limited attention to their role in NSSI. While some studies have observed elevated NSSI risk among youth with manic or mixed symptoms, such findings have largely been confined to bipolar-spectrum conditions [33]. The present study instead explores mixed features within the context of unipolar depressive episodes, specifically among adolescents and young adults. It is noteworthy that the detection rate of mixed features in our sample was considerably higher than that reported in previous DSM-5–based studies [34]. This discrepancy may be attributable to the composition of the sample, as the majority of participants were adolescents or young adults—a population typically regarded as being at elevated risk for mixed features [35]. With the progression of illness, some individuals within this group may subsequently convert to bipolar disorder, whereas others may evolve toward a relatively stable depressive disorder. By focusing on this population, which has hitherto received limited attention, the present study offers novel insights into the potential association between mixed features in depressive states and self-injurious behavior.

From a biological perspective, mixed features may reflect dysregulation within neurotransmitter systems, including dopamine and serotonin pathways, which are critically involved in affect regulation and impulse control [36, 37]. Psychologically, mixed features are characterized by emotional instability, heightened impulsivity, and behavioral activation, which may impair individuals’ capacity for effective emotion regulation [38, 39]. This dysregulation of emotional control may lead individuals to engage in NSSI as a maladaptive coping strategy aimed at alleviating negative affect. Indeed, emotional dysregulation has been identified as a core mechanism underlying NSSI, particularly among those with pronounced emotional instability [40]. Behavioral theories further suggest that NSSI recurrence is often maintained through negative reinforcement mechanisms [41, 42]. The behavioral activation inherent in mixed features may facilitate the use of NSSI as a means of immediate emotional relief, thereby perpetuating a maladaptive cycle that is resistant to disruption through conventional cognitive control processes [43, 44].

Clinically, these findings underscore the need to expand the identification of mixed features beyond their role in bipolar risk stratification to include sustained monitoring for self-injurious behavior. Notably, the presence of mixed features in depressed adolescents appears to confer heightened vulnerability to persistent, rather than episodic, NSSI. This highlights the importance of targeting the mechanisms—such as heightened emotional reactivity, impulsivity, and behavioral activation—that may maintain self-injurious behaviors over time. Recent studies have increasingly emphasized the need for personalized interventions in individuals with mixed features [45], given that standard treatments for unipolar depression may not adequately address this symptom profile [46]. Mechanism-based approaches, including emotion regulation training, impulsivity management, and behavioral modulation strategies, may be particularly relevant. In light of the chronic nature of NSSI in this subgroup, clinical care should prioritize early identification and sustained, individualized treatment planning.

This study possesses several notable strengths. First, it involves a relatively large sample drawn from a multicenter, prospective cohort, with a specific focus on youth depression, thereby enhancing the clinical relevance and applicability of the findings to this high-risk population. Second, missing data were rigorously addressed through both multiple imputation and complete-case analyses, which improved the robustness and reliability of the results. Third, the study employed multiple statistical models to differentiate risks for both incident and recurrent NSSI, allowing for a more nuanced understanding of the role of mixed features across distinct stages of NSSI risk. Nevertheless, several limitations warrant consideration. First, although diagnoses in this study were based on DSM-5 criteria, the absence of structured clinical interviews such as the SCID may have reduced diagnostic precision. Attention-deficit/hyperactivity disorder (ADHD) was not systematically assessed. Prior research indicates that ADHD increases the likelihood of mixed features among individuals with depression [47]. Undetected or mild ADHD therefore may have contributed to impulsivity or NSSI risk and introduced residual confounding. Other psychiatric comorbidities that are closely related to emotional dysregulation, including borderline personality disorder, eating disorders, and additional neurodevelopmental conditions, were also not formally evaluated because they were not included in the original study protocol. Although participants with severe impairments that prevented completion of the assessment were excluded, milder or undiagnosed comorbid conditions may have remained unrecognized. As a result, some residual confounding related to emotional dysregulation or unmeasured psychiatric conditions cannot be excluded. Second, childhood trauma and emotional dysregulation were not assessed in this cohort. Both constructs are strongly associated with NSSI, and the absence of these measures may have contributed additional residual confounding. Third, the six-month follow-up period may not be sufficient to capture the longer-term course of NSSI. Fourth, reliance on self-reported data for self-injurious behaviors introduces the possibility of recall bias. Fifth, several potentially important covariates, including medication use, psychological interventions, and family environment, were not assessed, which may limit the ability to fully account for factors influencing NSSI risk. Finally, the sample was recruited exclusively from South China. This geographic restriction may limit the generalizability of the findings to other regions or cultural contexts. Despite these limitations, the study provides novel longitudinal evidence regarding the contribution of mixed features to the persistence of self-injury in adolescents and young adults with depression.

In conclusion, while mixed features show limited predictive value for the initial onset of NSSI during follow-up, they demonstrate significant and independent prognostic relevance for NSSI recurrence. Importantly, this effect was consistent across different subgroups. These findings highlight the critical need to integrate mixed features into risk assessment and intervention strategies for self-injurious behaviors in adolescents and young adults with depression, especially among those exhibiting emotional instability and impulsivity. Future research should aim to extend follow-up periods, incorporate a wider array of clinical and environmental covariates, and elucidate the mediating mechanisms linking mixed features to NSSI, ultimately informing the development of personalized, mechanism-driven interventions.

## Supplementary Information

Below is the link to the electronic supplementary material.

Supplementary Material 1.

## Data Availability

Due to privacy restrictions related to patient data, the datasets generated and/or analyzed during the current study are not publicly available. However, they can be obtained from the corresponding author upon reasonable request.
