# Supplementary material for: Recurrent non-suicidal self-injury in depressed youth with mixed features: a 6-month prospective cohort study
Source: Child Adolesc Psychiatry Ment Health. 2025 Dec 9;20:4. doi: 10.1186/s13034-025-01006-z (PMC12801809; doi:10.1186/s13034-025-01006-z)
Supplement: Supplementary file 1 — Supplementary Material 1. [file 13034_2025_1006_MOESM1_ESM.docx]

Supplementary Materials: Missing Data Pattern of NSSI Assessments During Follow-Up in Depressed Youth


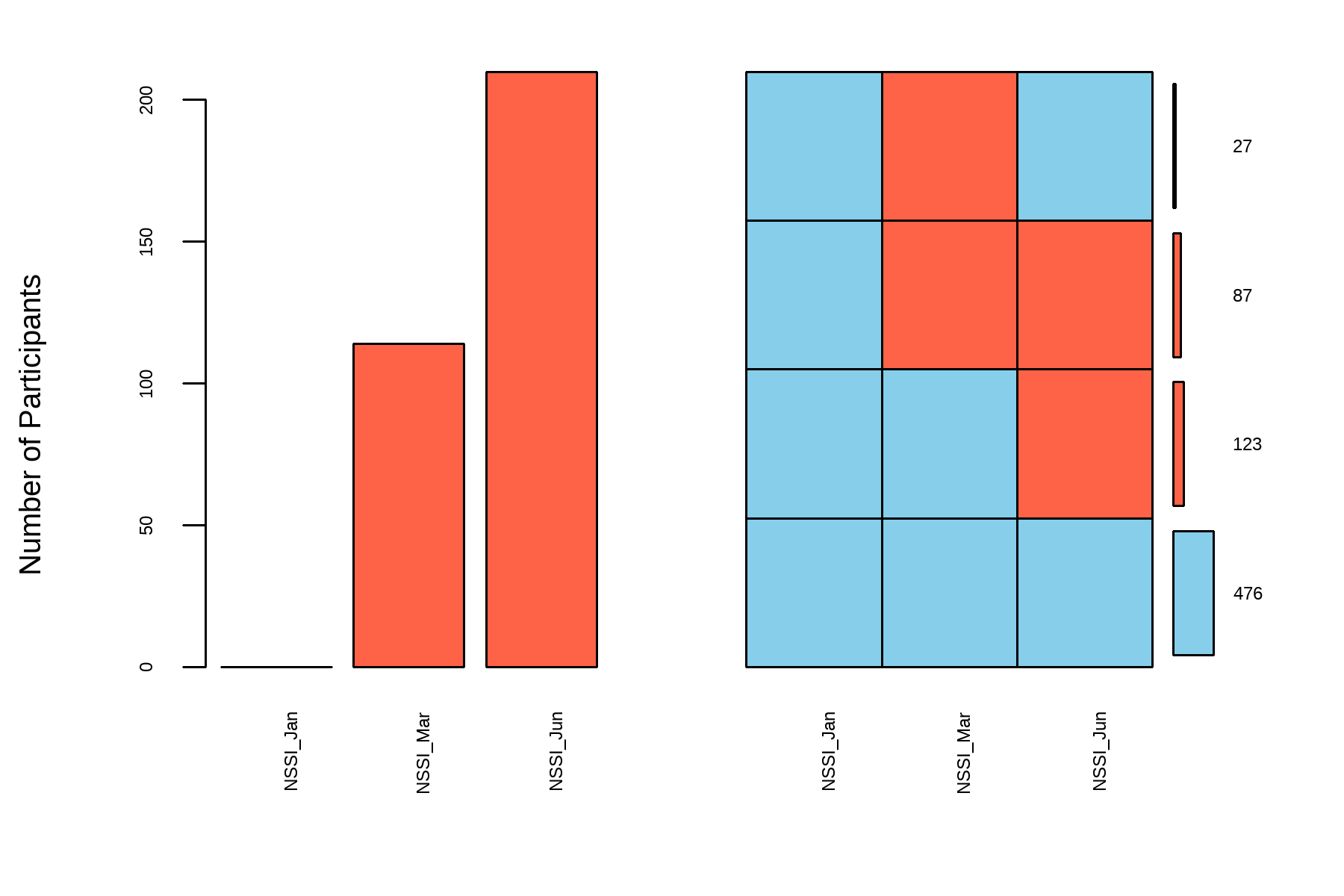


This figure shows the missing data pattern for non-suicidal self-injury (NSSI) assessments at three follow-up time points (1 month, 3 months, and 6 months) in adolescents and young adults with depression. Blue bars represent observed data, and red bars represent missing data. Numbers above each bar indicate the count of participants. The horizontal axis represents the NSSI assessments at each follow-up time point, and the vertical axis represents the number of participants.
